# Genetic Diversity of Sweetpotato (*Ipomoea batatas* (L.) Lam.) from Portugal, Mozambique and Timor-Leste

**DOI:** 10.3390/biology14111602

**Published:** 2025-11-15

**Authors:** Joana B. Guimarães, Maria Cristina Simões-Costa, Milton Pinho, Celina Maria Godinho, Paula Sá Pereira, João Neves Martins, Ana Ribeiro-Barros, Pedro Talhinhas, Maria Manuela Veloso

**Affiliations:** 1 Unidade de Investigação de Biotecnologia e Recursos Genéticos, Instituto Nacional de Investigação Agrária e Veterinária, Quinta do Marquês, 2784-505 Oeiras, Portugal; paula.sapereira@iniav.pt; 2 Linking Landscape, Environment, Agriculture and Food (LEAF Research Center), Instituto Superior de Agronomia, Universidade de Lisboa, Tapada da Ajuda, 1349-017 Lisboa, Portugal; simoescosta@isa.ulisboa.pt (M.C.S.-C.); nevesmartins@isa.ulisboa.pt (J.N.M.); ptalhinhas@isa.ulisboa.pt (P.T.); 3 Laboratório Associado TERRA, Instituto Superior de Agronomia, Universidade de Lisboa, 1349-017 Lisboa, Portugal; anaifribeiro@edu.ulisboa.pt; 4 Centro de Biotecnologia, Universidade Eduardo Mondlane, Maputo P.O. Box 257, Mozambique; milpter@gmail.com; 5 Faculdade de Ciências da Educação, Universidade Nacional Timor-Lorosa’e, R. Formosa 10, Dili P.O. Box 317, Timor-Leste; cellygodinho@gmail.com; 6 GREEN-it Bioresources4Sustainability, Instituto de Tecnologia Química e Biológica António Xavier, Universidade Nova de Lisboa, Avenida da República, 2780-157 Oeiras, Portugal; 7 Centro de Estudos Florestais, Instituto Superior de Agronomia, Universidade de Lisboa, 1349-017 Lisboa, Portugal

**Keywords:** plant germplasm, landraces, on-farm conservation, SSRs, genetic diversity, vitamin A deficiency, C-value, flow cytometry

## Abstract

Portugal contributed to the global diffusion of sweetpotato (*Ipomoea batatas* L. Lam.). Although it is of minor importance on the Portuguese mainland, it is one of the most common crops in the Azores and Madeira archipelagos and is highly relevant in the Portuguese ex-colonies Mozambique and Timor-Leste. We analyzed the genetic diversity and population structure of sweetpotato from these five geographic provenances using twelve nuclear simple sequence repeat (SSR) markers. We studied 45 accessions, 15 of which were collected from farmers’ fields in these five regions and 30 of which are held at “Banco de Germoplasma de Moçambique”. The SSR markers showed a high level of polymorphism and a high number of alleles per locus. Population structure analyses using Bayesian clustering (STRUCTURE) grouped accessions from farmers’ fields into two groups and divided samples of “Banco de Germoplasma de Moçambique” into three groups. A principal coordinate analysis (PCoA), based on the Bruvo distance, supported the population structure analysis. Concerning the genebank accessions, the two analyses indicated three clusters, all of them containing Mozambican landraces. From our results, it may be concluded that sweetpotato populations from the three countries do not share a common genetic background, despite the shared history of the countries.

## Simple Summary

Historically, Portugal played a role in the global diffusion of sweetpotato (*Ipomoea batatas*). Although, at present, the crop holds limited importance on the Portuguese mainland, it is still highly relevant in the Azores and Madeira archipelagos and in the Portuguese ex-colonies Mozambique and Timor-Leste. Using SSR markers, we assessed the genetic diversity of sweetpotato germplasm from a broad range of geographic regions from the three abovementioned countries, as well as the genetic diversity and identity of sweetpotato accessions held at “Banco de Germoplasma de Moçambique—IIAM”. The relationships and the genetic structures among sweetpotato accessions were also determined. The SSR analysis revealed high polymorphism and numerous alleles per locus, highlighting substantial genetic variability. Population structure analysis grouped on-farm accessions into two clusters, while the genebank samples formed three distinct clusters. Principal coordinate analysis based on the Bruvo distance supported these findings. The Azorean landraces were the most distant from all the other samples. Within the germplasm bank, Mozambican landraces were divided into three genetic clusters, reflecting diverse origins. Our results suggest that, despite shared historical ties, sweetpotato from Portugal, Mozambique and Timor-Leste do not share a common genetic background.

## 1. Introduction

Sweetpotato [*Ipomoea batatas* (L.) Lam., Convolvulaceae] is an allogamous self-incompatible root crop, which is clonally propagated, has a complex hexaploid (2n = 6x = 90) genome [1] and has an uncertain polyploid origin. Recently, Gao et al. [2] suggested that the hexaploid genome is best described as a segmental allohexaploid instead of being of a true allopolyploid or autopolyploid type.

Sweetpotato probably originated in Central America [3] and was domesticated at least 5000 years ago [4]. Columbus was responsible for sweetpotato introduction in Europe upon his return from America after this first maritime expedition (1492–1493). He left sweetpotato on São Miguel Island of the Azores Archipelago [5,6], from where it was rapidly dispersed to all the Azores Islands, Madeira Island and continental Portugal. A second introduction in continental Portugal from the Azores occurred by the time of the Second World War when a soldier named Lira took sweetpotato to the Algarve region (south of Portugal) [7].

Since Portugal was one of the earliest European empires to expand overseas, in the 15th century, sailors actively moved several crops, including sweetpotato, which was successfully introduced in the Old World and in Portuguese colonies (for instance, in Mozambique, Angola and Timor-Leste). Other sweetpotato dispersals occurred, and now it is widely cultivated globally.

In the Azores and Madeira archipelagos, sweetpotato is presently one of the most common crops, with economic, social and cultural importance; recently, 49 sweetpotato landraces were identified [8]. The most important on-farm cultivated landraces of Azores are “Roxa”, “Vermelha”, “Branca”, “Americana” and “da Madeira” [9]. On Madeira Island, the landraces “Barbiça”, “Cabreira”, “Carocha”, “Cinco Bicos” and “Inglesa” are considered subsistence crops, ranked second in root and tuber crop production [10]. The productivity and quality composition of the Madeira landraces “5 Bicos” and “Inglesa”, grown under different agroclimatic conditions, have already been studied [11]. In continental Portugal, a protected geographical indication (PGI) was assigned to “Lira”—“Batata-doce de Aljezur” in 2009 [12], while improved varieties with orange pulp, mainly obtained from the USA, have been introduced in Portugal in recent years.

After the Portuguese sweetpotato’s introduction in Africa, the crop spread inland [4]. Evidence indicates that due to this crop’s establishment in tropical Africa, the yam (Dioscorea species) was displaced and now is underutilized and considered an orphan crop [13]. In some areas of sub-Saharan Africa, sweetpotato ranks as the most widely grown root crop and provides household food security [14].

Most sweetpotato production in Africa is based on small or subsistence-level farmers, and in eastern Africa, this crop is known as “the protector of children” because just 125 g of fresh orange-fleshed sweetpotato (OFSP) roots contains enough beta carotene (vitamin A precursor) to provide the daily vitamin A needs of a pre-school-aged child [15]. It is recognized as a healthy food with disease-preventing properties [16] since, in addition to beta-carotene, it provides vitamins B1, B5, B6 and C as well as dietary fiber and minerals, e.g., potassium, magnesium, manganese and iron [17]. Vitamin A deficiency (VAD) is a significant problem not only in Africa but also in many developing countries, which reinforces the importance of the high beta-carotene content in OFSP [18].

In Mozambique, about 60,930 ha of sweetpotato is cultivated yearly [19], and sweetpotato production has doubled due to an important OFSP breeding program directed at farmers in southern Mozambique [20,21].

In the Southeastern Pacific, the crop was introduced from Ecuador or Peru, circa 1000 years ago, by Polynesian voyagers before its introduction by European (Portuguese and Spanish) settlers, probably in the early 19th century [22,23]. In Timor-Leste, which ranked third as a UN country with a high percentage of chronically malnourished children, sweetpotato is also an important staple food, although its national productivity is very low [24]. In this country, the Seeds of Life program aimed to increase the productivity of sweetpotato by introducing germplasm from CGIAR centers and from neighboring countries, such as Indonesia and the Philippines [25]. In 2016, with the support of the Australian Centre for International Agriculture Research, two yellowish sweetpotato varieties were launched in Timor-Leste. Twenty-five distinct local cultivars obtained from three markets in Dili were phenotypically characterized [26], with the aim of safeguarding the local sweetpotato genetic resources. A strategy for sweetpotato conservation was defined [26] using 20 morphological descriptors [27], and considerable diversity was found.

There is a global effort to study the existing diversity in farmers’ fields [28], not only at the morphological level but also at the molecular level. Germplasm collections held in genebanks have also been characterized using molecular markers [16,29,30]. However, such studies concerning sweetpotato from Portugal, Mozambique and Timor-Leste have not been performed, which is the subject of our work. Indeed, the genetic diversity of sweetpotato from mainland Portugal has neither been studied nor compared to that of the Azores and Madeira archipelagos. In Mozambique, the genetic diversity of on-farm sweetpotato has also never been studied. However, genebank accessions have been studied by Maquia et al. [31], using RAPD markers, and by Pinho Master Thesis [32], using SSRs. We used Pinho experimental data in the present work, using the POLYSTAT program for statistical analysis, which takes into consideration the hexaploidy nature of sweetpotato.

Considering the global importance of sweetpotato, it is of great relevance to study the existing relationship between accessions from different regions. Thus, our study covers the aforementioned insufficiently explored regions using SSR markers, which fills the gap in research on the genetic structures and relationships of sweetpotato across regions.

Using SSR markers, we (i) assessed the genetic diversity of sweetpotato germplasm collected from farmers’ fields in a broad range of geographic regions; (ii) assessed the genetic diversity and identity of 30 sweetpotato accessions held at “Banco de Germoplasma de Moçambique—IIAM” in Maputo, Mozambique; and (iii) determined the relationships and the genetic structures among the several sweetpotato accessions.

## 2. Materials and Methods

### 2.1. Plant Material

A total of 45 accessions were studied: 15 samples were collected from farmers’ fields in 3 countries (Portugal, Mozambique and Timor-Leste), and 30 accessions are held at “Banco de Germoplasma de Moçambique—IIAM” in Maputo (Table 1).

### 2.2. DNA Extraction, PCR Amplification and Fragment Sizing

DNA was isolated from young leaves using the innuPREP Plant DNA Kit (Analytik Jena AG, Berlin, Germany), according to the manufacturer’s protocol. DNA quality and concentration were visually checked on 0.8% agarose gel. DNA concentration was also estimated using a NanoDrop ND2000 spectrophotometer (Thermo Scientific, Waltham, MA, USA). All accessions were genotyped twice (technical replicates).

### 2.3. Genetic and Genomic Analysis

Genotyping was based on 12 SSR loci (8 were developed by Buteler et al. [33] from a genomic library of *I. batatas*, and 4 were referenced by Karuri et al. [34]). The primer sequences are provided in Table S1.

Amplification was performed in a final volume of 25 μL containing 20 ng of DNA, 0.25 µM forward and reverse primers and 12.5 μL of DreamTaq PCR master mix (ThermoFisher, Waltham, MA, USA). Each PCR was programmed as follows: initial denaturation for 3 min at 94 °C, followed by 30 cycles of denaturation at 94 °C for 30 s, annealing at optimum Ta for 30 s and extension at 72 °C for 1 min. A final extension step was performed at 72 °C for 7 min, and the reaction was finished with a continuous cycle at 4 °C. The reactions were conducted in a MyCycler Thermal Cycler (BIO-RAD, Catalog #170-9703, Hercules, CA, USA) and carried out separately for each microsatellite.

Amplification products were separated via polyacrylamide gel electrophoresis (PAGE) performed with a 4% stacking gel and 12% running gel at 200 V and 35 mA until the end of the run, and these products were further visualized by staining the gel with ethidium bromide (0.5 g µg/mL) for 20–30 min. Images were captured using Gel Doc-XR (BIO-RAD, Hercules, CA, USA), and the gel was analyzed using Image Lab Software 4.1 (BIO-RAD, Hercules, CA, USA). The molecular weight of DNA fragments was determined using the software Quantity One 1-D (BIO-RAD, Hercules, CA, USA). The DNA ladder “HyperLadder^TM^ 25 bp” (BIO-33057, Bioline, Heidelberg, Germany), with a pattern of 12 regularly spaced bands, ranging from 25 bp to 500 bp, was used to infer the size of the DNA in the sample lanes.

The genome size of the sweetpotato from farmers’ fields was estimated through applying flow cytometry (FCM). Suspensions of intact nuclei were prepared for analysis following the method of Galbraith et al. [35]. Each nuclear suspension was sieved through a 30 μm nylon mesh to remove large debris, and the obtained nuclei were stained with 25 μg mL^−1^ of propidium iodide (PI; Sigma-Aldrich, St. Louis, MO, USA). Numeric data and fluorescence graphs were acquired using Sysmex FloMax software v2.4d (Sysmex, Görlitz, Germany), as described by Guillengue et al. [36]. From the analyzed samples, the quantity of DNA (in pg, per nucleus) was estimated. Tomato (*Solanum lycopersicum* L.; 2C = 1.96 pg) and soybean (*Glycine max* L.; 2C = 2.45 pg) were used as internal standards [37,38].

### 2.4. Data Analysis

A mixture of inheritance patterns exists within sweetpotato, which can be a problem in determining allelic configurations when using molecular markers [39].

Frequently, molecular data are “diploidized” because it is not possible to determine the allele dosage [16]. The programs created for data analysis and genetic population studies of polyploidy still face challenges [40,41].

In this study, microsatellite data were analyzed using the R package POLYSAT version 1.7 [42], which is a program for importing and exporting data to other software. Specialized genetic distances, like the Bruvo distance and those obtained using principal coordinate analysis (PCoA), were calculated using this package. The distance matrix was exported from POLYSAT for further analysis. The Bruvo distance is a measure of genetic differentiation specific to microsatellite data that allows for missing data and takes into account different numbers of repeat lengths and numbers of alleles per locus [43].

The neighbor-joining algorithm, implemented in the DARwin software package version 6.0.12 [44], was based on a dissimilarity matrix, and the reliability of the tree topology was assessed via bootstrapping over 1000 replicates.

The software POLYGENE v1.2 [45] was used to estimate, for each locus, the following parameters: the observed number of alleles “Na”, the effective number of alleles “Ne”, observed heterozygosity “Ho”, expected heterozygosity “He”, Shannon’s Information Index “I”, polymorphism information content “PIC” and the inbreeding coefficient “Fis”.

The level of genetic stratification was assessed using STRUCTURE v.2.3.4 software [46], considering both the admixture model and the correlated allele frequencies between populations, with values of K set from 1 to 5. Population information was incorporated into the analyses (LOCPRIOR model). Each run consisted of a burn-in period of 50,000 steps followed by 1,000,000 MCMC (Monte Carlo Markov Chain) replicates. K is the probable maximum population number that is assumed to represent and contribute to the genotypes of sampled individuals. To check the consistency of the results between runs with the same K value, five replicates were run for each assumed value. The approach suggested by Evanno et al. [47] was adopted to calculate the most likely value of K based on the second-order rate of change in the likelihood function with respect to K (ΔK). Once the number of genetic clusters was established, each individual was assigned to a cluster, and the overall membership of each sampled individual in each cluster was estimated.

## 3. Results

### 3.1. Genetic Diversity in the Sweetpotato Germplasm

The SSR primer pairs amplified 103 alleles for the on-farm accessions, with a mean value of 11.4 alleles, ranging from 6 (IBCIP13) to 19 (IBR19), and with a mean effective number of alleles “Ne” of 5.68. The I value for each SSR ranged from 1.24 to 2.73, and the diversity “He” ranged from 0.54 to 0.92 (Table 2).

For the genebank accessions, 122 alleles were amplified, with a mean value of 12.2 alleles, ranging from 6 (IBR16) to 19 (IB248), and with an effective number of alleles “Ne” of 8.10. The I value for each SSR ranged from 1.56 to 2.82, and the diversity (He) ranged from 0.75 to 0.90 (Table 3).

The diversity parameters Ho and He, tested across microsatellite markers, showed that Ho was significantly different between the accessions from farmers’ fields and the genebank, being higher for the genebank samples. The highest values of Ne and He were displayed by the loci IBR19 (on-farm accessions) and IB248 (genebank), while the lowest values were displayed by the loci IB255F1 (on-farm) and IBR16 (genebank) (Table 2 and Table 3). The SSR screening of the on-farm and genebank accessions is shown in Tables S2 and S3. For both analyses, the polymorphism information content “PIC” indicated that the used loci were useful diversity indicators.

Great differentiation (F_ST_) was observed among the on-farm accessions (Table 2).

### 3.2. Phylogenetic Analysis and Principal Coordinate Analysis

**Figure 1:**
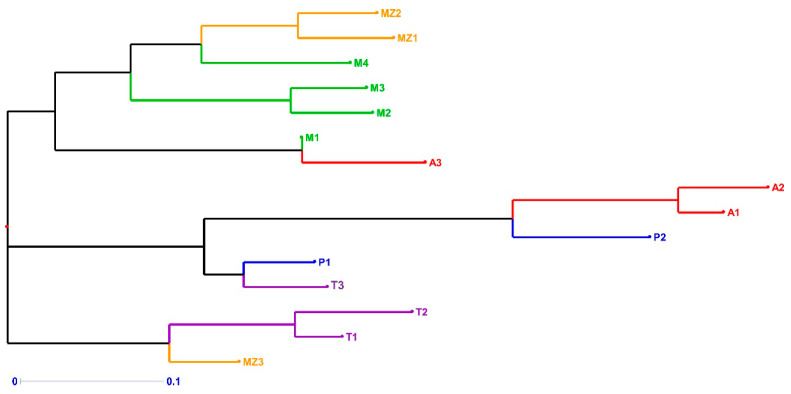
Neighbor-joining dendrogram of 15 on-farm accessions, identified by nine SSR markers. Branch lines represent individual accessions [red: Azores Islands (A); yellow: Mozambique (MZ); green: Madeira Island (M); blue: continental Portugal (P); purple: Timor-Leste (T)].

**Figure 2:**
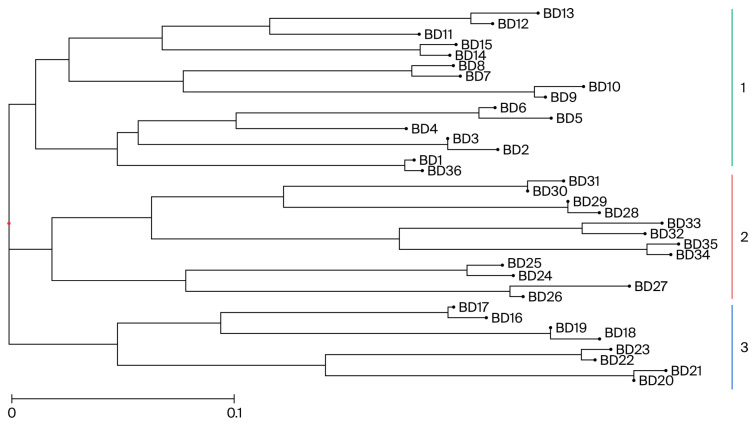
Neighbor-joining dendrogram of 30 genebank accessions, identified by ten SSR markers. Branch lines represent individual accessions.

The neighbor-joining trees generated from the Bruvo genetic distance and constructed using DARwin software version 6 [44] are shown in Figure 1 and Figure 2. For the accessions collected from farmers’ fields, a clear distribution pattern of the samples in three groups was found (Figure 1): 1—all the Madeiran accessions, the Mozambican breeding varieties MZ1 and MZ2 and the Azorean accession A3; 2—all the continental Portuguese accessions, the Azorean accessions A1 and A2 and T3 from Timor-Leste; 3—T1 and T2 from Timor-Leste and the breeding variety MZ3 from Mozambique.

For the genebank samples, the corresponding neighbor-joining tree shows a clear separation of the 30 accessions into three different groups (Figure 2): the first group includes Mozambican landraces [“UNK Malawe” (BD4), “Chissicuene 3” (BD9), “Nhacutze 3” (BD11), “Nhacutze 4” (BD15) and “Admarc” (BD36)] and breeding varieties [“Gloria” (BD1), “Lourdes” (BD5), “Namanga” (BD6), “Esther” (BD7) and “Bela” (BD8)], as well as modern cultivars [“Naspot 5” (BD3), “Jonathan” (BD12) and “Maphuta” (BD14) from Uganda, Peru and South Africa, respectively]; the second group includes Mozambican landraces [“Nwamazamba” (BD23), “Chitandzana” (BD26), “Xiadlaxakan” (BD29), “Xiphone” (BD30) and “Nwamonguase” (BD32)], Mozambican breeding varieties [“Irene” (BD27) and “Tio Joe” (BD34)], the Kenian landrace “SPK004” (BD31), the breeding clone “LO323” (BD33) from the USA and the “Atacama” (BD25) South African modern cultivar; the third group is composed of the Mozambican landrace “Canassumana” (BD20), the Mozambican breeding varieties “Erica” (BD19) and “Sumaia” (BD22) and the modern cultivar “Resisto” (BD18) from the USA.

**Figure 3:**
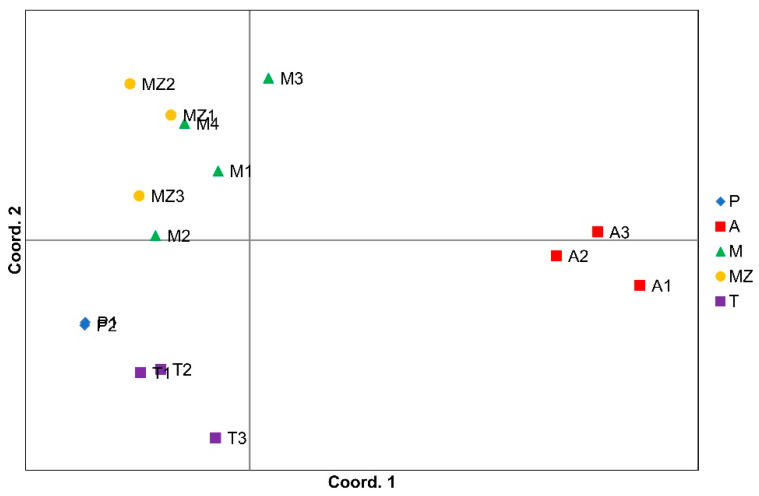
Principal coordinate analysis (PCoA) of SSR markers showing allelic variation among accessions collected from farmers’ fields. Symbols represent individual accessions [red: Azores Islands (A); yellow: Mozambique (MZ); green: Madeira Island (M); blue: continental Portugal (P); purple: Timor-Leste (T)].

In order to assess the relationships among the accessions and how they cluster based on the SSR results, a principal coordinate analysis (PCoA) was performed. The samples from farmers’ fields are distributed into three groups (Figure 3). The Azorean landraces (A) are the most distant from all the other populations. Mozambican (MZ) and Madeiran (M) accessions are on the upper left side of the graph, while samples from Timor-Leste (T) and continental Portugal (P) are in the lower left part of the graph. All the samples, except the Azorean ones, have similar values at coordinate 1 but markedly different values at coordinate 2.

**Figure 4:**
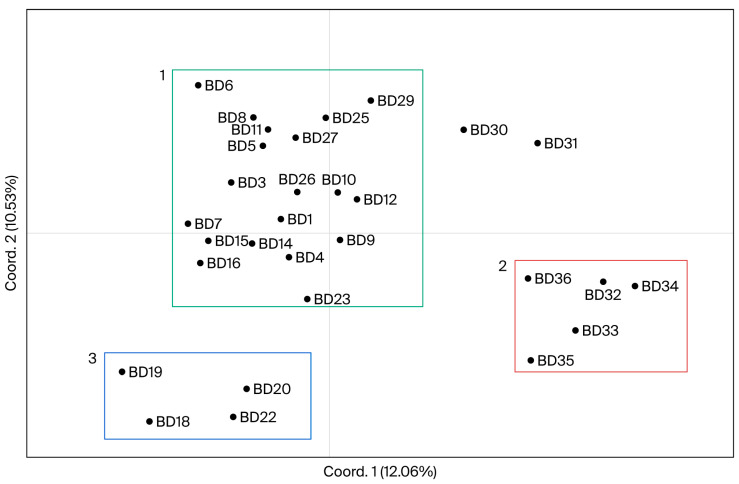
Principal coordinate analysis (PCoA) of SSR markers showing allelic variation among 30 accessions held at “Banco de Germoplasma de Moçambique”.

For the genebank accessions, the first two principal coordinates account for 10.53% and 12.06% of molecular variation, respectively, which discriminates 30 accessions in two dimensions. Although the samples are scattered in the graph, we can consider that the total accessions are grouped into three clusters (Figure 4). A total of 63% of the samples are found in the upper and central left parts of the graph, 37% of which includes Mozambican landraces [“UNK Malawe” (BD4), “Chissicuene 3” (BD9), “Nhacutze 3” (BD11), “Nhacutze 4” (BD15), “Nwamazamba” (BD23), “Chitandzana” (BD26) and “Xiadlaxakau” (BD29)] and 63% of which comprises breeding varieties [“Gloria” (BD1), “Lourdes” (BD5), “Namanga” (BD6), “Esther” (BD7), “Bela” (BD8) and “Irene” (BD27)], modern cultivars [“Naspot5” (BD3), “Jonathan” (BD12), “Maphuta” (BD14) and “Atacama” (BD25)] and two samples from Angola (BD10 and BD16). On the lower left side of the graph are the Mozambican breeding varieties “Erica” (BD19) and “Sumaia” (BD22), clustered with the Mozambican landrace “Canassumana” (BD20) and the modern cultivar “Resisto” (BD18) from the USA. On the lower right side of the graph, the Mozambican landraces “Nwamonguase” (BD32) and “Admarc” (BD36) are clustered with the breeding varieties “LO323” (BD33), “Tio Joe” (BD34) and “MGCL-01” (BD35). The landraces “Xiphone” (BD30—Mozambique) and “SPK004” (BD31—Kenya) in the upper right part of the graph do not cluster with any group.

### 3.3. Population Structure of Sweetpotato Germplasm

**Figure 5:**
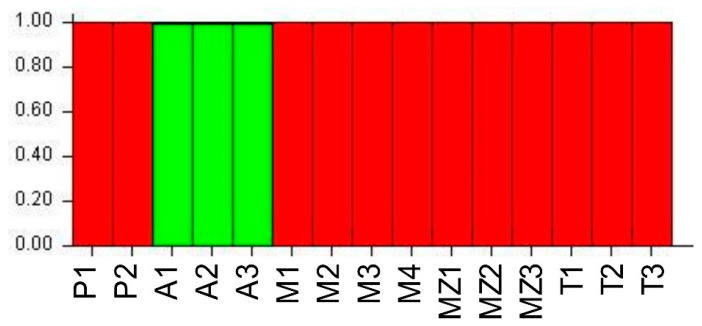
The population structure of accessions from farmers’ fields. SSR marker data was estimated using the model-based Bayesian algorithm implemented in the STRUCTURE program. This figure shows the proportion of assignment of individuals to K = 2. Each accession is represented by a vertical line. A—“Azores Islands”; M—“Madeira Island”; MZ—“Mozambique”; P—“continental Portugal”; T—“Timor-Leste”.

The Bayesian approach, corresponding to accessions collected from farmers’ fields, indicated that the most likely number of genetic clusters is K = 2 (ΔK = 767) (Figure S1). Based on the results of the STRUCTURE analysis, the two groups assigned at K = 2 correspond to landraces from the Azores Islands (green color), and all the other accessions are represented by red (Figure 5).

The Bayesian approach for the genebank accessions indicated that the most likely number of genetic clusters is K = 3 (ΔK = 5.741) (Figure S2). At K = 3, the clustering of sweetpotato samples is represented by three colors. The Mozambican samples are spread across three subgroups (30% green, 17% blue and 17% red).

**Figure 6:**
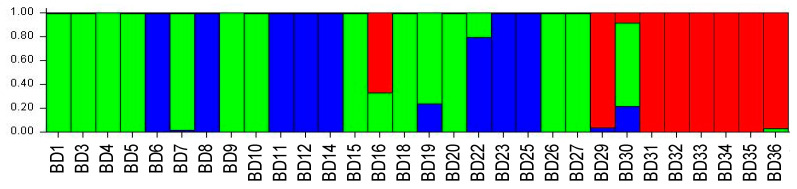
The structure of 30 accessions held at “Banco de Germoplasma de Moçambique”, using SSR marker data as estimated using the model-based Bayesian algorithm implemented in the STRUCTURE program. This figure shows the proportion of assignment of individuals to K = 3 sub-population groups. Each accession is represented by a vertical line.

The green cluster includes Mozambican landraces [“UNK Malawe” (BD4), “Chissicuene 3” (BD9), “Nhacutze 4” (BD15), “Canassumana” (BD20) and “Chitandzana” (BD26)] and breeding varieties [“Gloria” (BD1), “Lourdes” (BD5), “Esther” (BD7) and “Irene” (BD27)], as well as modern cultivars [“Naspot 5” (BD3) from Uganda and “Resisto” (BD18) from the USA]. In the blue group are the Mozambican landraces “Nhacutze 3” (BD11) and “Nwamazamba” (BD23) and breeding varieties “Namanga” (BD6), “Bela” (BD8) and “Sumaia” (BD22), as well as the modern cultivars “Jonathan” (BD12) from CIP and “Maphuta” (BD14) and “Atacama” (BD25) from South Africa. The red group includes Mozambican [“Xiadlaxakau” (BD29), “Nwamonguase” (BD32) and “Admarc” (BD36)] and Kenyan [“SPK004” (BD31)] landraces and the breeding varieties “LO323” (BD33), “Tio Joe” (BD34) and “MGCL-01” (BD35). The Mozambican landrace “Xiphone” (BD19) and breeding variety “Erica” (BD30) and the Angola sample “Cacuso” (BD16) are considered to be admixed (Figure 6).

### 3.4. Cytogenomic Results

The nuclear DNA content determined ranged between 3.00 pg/2C [“Batata Doce Regional” (A1), a landrace from the Azores Islands] and 3.50 pg/2C [“Lira” (L1), a landrace from continental Portugal] (Table 4).

## 4. Discussion

The goal of this study was to understand the diversity patterns of sweetpotato of three interrelated countries, Portugal, Mozambique and Timor-Leste. Landraces grown in farmers’ fields as well as accessions held at “Banco de Germoplasma de Moçambique” were analyzed. Landrace diversity is still high in Portugal, a country considered a landrace hotspot [49]. On the continent, as well as in the Azores and the Madeira archipelagos, several sweetpotato landraces are still maintained on farms, and accessions are conserved ex situ in the ISOPLEXIS germplasm bank [8]. However, landraces in farmers’ fields have decreased globally due to young people’s migration to urban areas where the perceived quality of life is higher.

In Africa, populations prefer white- and cream-fleshed sweetpotato landraces, but erosion is observed due to the cultivation of more nutritious OFSP varietal clones, for example, the Gloria and the Ininda OFSP in Mozambique [20]. The effort to change people’s preference for more nutritious OFSP is stimulated by CIP [50], and the Mozambican Institute of Agricultural Research (IIAM) is also participating in this goal since OFSP consumption will combat chronic vitamin A deficiency. Importantly, the Resisto modern cultivar from the USA has been heavily used as an OFSP parent in East African breeding programs [51]. Nevertheless, it should be remembered that landraces can harbor rare alleles and unusual allele combinations, which, in breeding programs, can provide important clues regarding adaptation to climate change, pests, diseases and consumer preferences [52]. Since only a relatively small number of high-yield, genetically uniform varieties are used in modern breeding programs, it is beneficial to conduct germplasm collection missions, in order to safeguard the remaining genetic diversity and agronomic potential. This is a critical effort for providing the genetic materials needed to further ensure efficient plant breeding. The IIAM is already maintaining Mozambican sweetpotato landraces at “Banco de Germoplasma de Moçambique” (Table 1B), which is of major importance considering that the majority of accessions in international germplasm banks are instead cultivars or breeding lines [30].

In Timor-Leste, a phenotypic characterization of local varieties was recently published [26], but there is no information on landrace relationships to the geographic regions of the country.

When we aimed to genetically characterize the sweetpotato accessions using SSR markers, we faced the difficulty of scoring the alleles in some chromatograms. Low-quality genotyping patterns due to uncertain allele dosages and the possibility of the non-random inheritance of alleles have also been reported [53]. This problem results from the existence of a mixture of inheritance patterns in sweetpotato [39]. Despite this, in our study, the SSR markers used showed a high level of polymorphism (Table 2 and Table 3). A study of 119 Latin American accessions using six primers [3] and a study of 57 cultivars of East Africa using four primers [54] were also able to identify a high level of polymorphism. Comparable values were also reported when studying West African sweetpotato [55].

We detected a higher number of alleles per locus than those reported by Karuri et al. [34], Veasey et al. [56] and Xin-Sun et al. [57] for 89 Kenyan genotypes, 78 Brazilian accessions collected from 19 local farmers’ fields and 380 Chinese sweetpotato accessions from different agroclimatic zones, respectively. Since different sets of markers were used in these studies, we should be cautious about the values reported because microsatellite type and the number of repetitions affect the number of alleles and diversity assessment [58].

When analyzing the PCoA and the STRUCTURE results of the Portuguese accessions, we unexpectedly found that the Azores accessions have no relationship with those of continental Portugal and Madeira Island (Figure 3 and Figure 5). We explain this observation by the fact that, in our work, we only studied sweetpotato from one of the Azores Islands (i.e., Terceira Island). It was also surprising to find that, in the PCoA diagram, the Madeiran sweetpotato landraces cluster together with the Mozambican varietal clones, which results from a breeding program using genetic material from Peru and the USA. Our hypothesis is that the Madeiran accessions we studied may already be the result of introductions additional to those of Columbus’s time. In East Africa, two separate sweetpotato genepools have been introduced, one by the Portuguese and another by the British later on [59]; this may explain the similarity between sweetpotato accessions from Madeira Island and Mozambique.

An analysis of ex situ accessions held at “Banco de Germoplasma de Moçambique” (Table 1B) revealed that all the landraces from the Gaza province (BD11, BD15, BD23, BD26, BD29) cluster in the same PCoA group (Figure 4). However, with the Bayesian approach, no specific association with geographic localization is seen (Figure 6). A similar observation was reported by Rodriguez-Bonilla et al. [28], Tumwegamire et al. [51], Gwandu et al. [54] and Elameen et al. [59], who found that genotypes collected in a given region often displayed molecular marker variability similar to that observed over the entire sampled area. This possibly results from farmer activities using the extensive exchange of materials.

For the eight Mozambican OFSP cultivars BD1, BD5, BD6, BD7, BD8, BD22, BD27 and BD 34 (Table 1B), no association with geographic regions was found using either the PCoA (Figure 4) or the Bayesian approach (Figure 6). This fact can be explained by the breeding program used in Mozambique. Although the country has several agroecosystems [60], according to Andrade et al. [20], the breeding objective is to obtain high-yield cultivars for the whole country, instead of niche breeding cultivars. Examples are the BD1 and BD7 cultivars, which are selected for Angónia (R10 agroecological zone; average rainfall, 1200 mm/year) and Chókwè (R2 agroecological zone, semi-arid area; average rainfall, 623 mm/year), respectively, but are cultivated by farmers in other regions.

We should emphasize the importance of genebanks and the characterization of their accessions in order to detect material useful for developing varieties adapted to environmental stress and resistance/tolerance to pests and/or diseases. Consumer preferences and nutritional content are also factors to consider. The integration of genomic selection into breeding is an important contribution to increasing the productivity and nutritional value of sweetpotato [50]. Microsatellite markers are highly efficient, but they could have some limitations when used for polyploid species. Thus, it is advisable to use other markers, such as those not available at the time of our study. One example is the recently developed optimized genotyping-by-sequencing for highly heterozygous and polyploid genomes (GBSpoly) [29,53]. A further phenotypic and molecular characterization of sweetpotato germplasm will provide valuable information for breeders, which can increase and improve food supply and nutritional value, mainly for people living in developing countries.

## 5. Conclusions

SSRs were efficient markers in discriminating all the sweetpotato accessions.

Considering that on-farm conservation is a relevant strategy to maintain the evolutionary forces within and between components of the agricultural system, it is beneficial to conduct germplasm collection missions targeted toward sweetpotato landraces to safeguard their diversity and agronomic potential. Special attention should be given to Timor-Leste, where studies are rudimentary and particularly recent.

Additional research is needed in order to better understand the relationships of sweetpotato from three countries with historic links: Portugal, Mozambique and Timor-Leste. The number of landraces to be analyzed should be increased in order to determine the existing levels of diversity within these landraces. After acquiring such information, a suitable on-farm conservation strategy can be established.

## Figures and Tables

**Table 1:** Sweetpotato (*Ipomoea batatas*) sampling locations. (**A**) Collected from farmers’ fields. (**B**) Held at “Banco de Germoplasma de Moçambique”.

**(A)**						
**Accession**	**Type**	**Country**	**Sampling Location**	**Latitude;** **Longitude**	**Traditional Name**	**Flesh** **Color**
P1-Li 01	Landrace	Portugal—mainland, Europe	Lourinhã	39°13′55.9″ N; 9°12′14.7″ W	Lira	Yellow
P2-Li 02	Landrace	Portugal—mainland, Europe	Lourinhã	39°13′55.9″ N; 9°12′14.7″ W	Lira	Yellow
M 01	Landrace	Portugal—Madeira Island, Europe	Porto Moniz	32°51′50.256″ N; 17°10′15.887″ W	Branca de Cinco Bicos	White
M 02	Landrace	Portugal—Madeira Island, Europe	Porto Moniz	32°51′36.627″ N; 17°10′9.854″ W	Amarela—Sítio Santo	Yellow
M 03	Landrace	Portugal—Madeira Island, Europe	Ponta do Pargo	32°48′40.932″ N; 17°14′55.031″ W	Inglesa	White
M 04	Landrace	Portugal—Madeira Island, Europe	São Vicente	32°47′20.026″ N; 17°2′9.24″ W		White
A1	Landrace	Portugal—Azores Islands, Europe	Fontinhas	38°45′3.424″ N; 27°6′40.723″ W	Batata Doce da Madeira	White
A2	Landrace	Portugal—Azores Islands, Europe	Fontinhas	38°44′35.02″ N; 27°6′28.008″ W	Batata Doce Roxa	Cream
A3	Landrace	Portugal—Azores Islands, Europe	Fontinhas	38°44′35.02″ N; 27°6′28.008″ W	Batata Doce Branca	Cream
MZ1-Gl 03	Cultivar	Mozambique, Africa	Angónia	14°55′47.852″ S; 34°11′35.092″ E	Gloria	Deep orange
MZ2-Gl 04	Cultivar	Mozambique, Africa	Angónia	14°55′47.852″ S; 34°11′35.092″ E	Gloria	Deep orange
MZ3-In 05	Cultivar	Mozambique, Africa	Angónia	14°55′47.852″ S; 34°11″35.092″ E	Ininda	Dark Orange
T 01	Landrace	Timor-Leste, Asia	Dili, Timor-Leste		Vermelho Laranja	Orange
T 02	Landrace	Timor-Leste, Asia	Dili, Timor-Leste		Vermelho Branco	White
T 03	Landrace	Timor-Leste, Asia	Dili, Timor-Leste		Amarelo Rosada	Yellow
(**B**)						
**Accession**	**Type**	**Country/Country Region**	**Sampling Location**	**IIAM Genebank Number**	**Traditional Name**	**Flesh** **Color**
BD1	Cultivar **	Mozambique, Africa	Angónia	1	Gloria	Orange
BD3	Modern Cultivar *	Uganda, Africa		3	Naspot 5	Orange
BD4	Landrace *	Mozambique, Africa	Zambezia	4	UNK—Malawe	Yellow
BD5	Cultivar **	Mozambique, Africa	Maputo	5	Lourdes	Orange
BD6	Cultivar **	Mozambique, Africa	Maputo	6	Namanga	Orange
BD7	Cultivar **	Mozambique, Africa	Maputo	7	Esther	Orange
BD8	Cultivar **	Mozambique, Africa	Maputo	8	Bela	Orange
BD9	Landrace *	Mozambique, Africa	Inhambane	9	Chissicuane 3	White
BD10		Angola, Africa	Angola	10	Gaba Gaba	Orange
BD11	Landrace *	Mozambique, Africa	Gaza	12	Nhacutse 3	Cream
BD12	Modern Cultivar *	Peru, South America	CIP—Peru	13	Jonathan	Orange
BD14	Modern Cultivar	South Africa, Africa	South Africa	16	Mafuta	Yellow
BD15	Landrace *	Mozambique, Africa	Gaza	17	Nhacutse 4	White
BD16		Angola, Africa	Angola	18	Cacuso	White
BD18	Modern Cultivar *	USA, North Aerica	USA	20	Resisto	Orange
BD19	Cultivar **	Mozambique, Africa	Maputo	21	Erica	Orange
BD20	Landrace *	Mozambique, Africa	Zambézia	22	Canassumana	Yellow
BD22	Cultivar **	Mozambique, Africa	Maputo	25	Sumaia	Orange
BD23	Landrace *	Mozambique, Africa	Gaza	26	Nwamazambane	Yellow
BD25	Modern Cultivar *	South Africa, Africa	South Africa	28	Atacana	Yellow
BD26	Landrace *	Mozambique, Africa	Gaza	29	Xitan dzana	Orange
BD27	Cultivar **	Mozambique, Africa	Mozambique	30	Irene	Orange
BD29	Landrace *	Mozambique, Africa	Gaza	34	Xiadla xa kau	Yellow
BD30	Landrace *	Mozambique, Africa	Inhambane	35	Xiphone	White
BD31	Landrace *	Kenya, Africa	Kenya	36	SPK 004	Orange
BD32	Landrace *	Mozambique, Africa	Gaza	37	Nwa mongoane	Cream
BD33	Breeding Clone *	Peru, South America	CIP—Peru	38	Lo323	Orange
BD34	Cultivar **	Mozambique, Africa	Maputo	40	Tio Joe	Orange
BD35	Landrace *	Mozambique, Africa	Angónia	41	MGCL-01	Orange
BD36	Landrace *	Mozambique, Africa	Zambézia	43	Admarc	Cream

* According to Andrade et al. 2016 [14]; ** according to Andrade et al. 2017 [20].

**Table 2:** The genetic diversity of the 15 accessions from the 5 on-farm sweetpotato populations across the SSR loci. Na—number of alleles; Ne—effective number of alleles; Ho—observed heterozygosity; He—expected heterozygosity; PIC—polymorphism information content; I—Shannon information index; F—inbreeding coefficient; F_ST_—differentiation index.

Locus	Na	Ho	He	PIC	Ne	I	F	F_ST_
IBS07	13.00	0.40	0.78	0.77	4.63	1.99	0.49	0.20
Ib255F1	8.00	0.09	0.54	0.52	2.15	1.24	0.83	0.35
Ib242	8.00	0.49	0.73	0.71	3.76	1.64	0.33	0.20
IBR16	7.00	0.55	0.67	0.63	3.00	1.41	0.18	0.13
Ib318	15.00	0.67	0.87	0.86	7.99	2.35	0.23	0.15
Ib297	15.00	0.63	0.90	0.89	9.88	2.49	0.30	0.20
IBR19	19.00	0.60	0.92	0.91	12.54	2.73	0.35	0.17
Ib286	12.00	0.48	0.77	0.75	4.43	1.89	0.39	0.10
IbCIP13	6.00	0.12	0.63	0.58	2.73	1.28	0.81	0.42
Mean	11.4	0.45	0.76		5.68	1.89	0.43	0.21

**Table 3:** The genetic diversity of the 30 sweetpotato accessions held at “Banco de Germoplasma de Moçambique—IIAM” across the SSR loci. Na—number of alleles; Ne—effective number of alleles; Ho—observed heterozygosity; He—expected heterozygosity; PIC—polymorphism information content; I—Shannon information index; F—inbreeding coefficient.

Locus	Na	Ho	He	PIC	Ne	I	F
IB-S07	8.00	0.10	0.78	0.74	4.44	1.70	0.87
Ib-255F1	18.00	0.30	0.89	0.88	9.05	2.50	0.66
Ib-248	19.00	0.10	0.93	0.93	15.25	2.82	0.89
Ib-242	10.00	0.36	0.79	0.76	4.74	1.82	0.54
Ib-316	13.00	0.18	0.90	0.89	9.57	2.41	0.80
IB-R16	6.00	0.00	0.75	0.72	4.02	1.56	1.00
Ib-318	8.00	0.12	0.81	0.79	5.36	1.82	0.85
Ib-297	13.00	0.12	0.89	0.88	9.18	2.36	0.87
IB-R19	12.00	0.08	0.89	0.88	9.00	2.31	0.91
Ib-286	15.00	0.52	0.90	0.90	10.34	2.48	0.42
Mean	12.2	0.19	0.85		8.10	2.18	0.78

**Table 4:** Genotypes of *I. batatas* collected from farmers’ fields and mean DNA content determined by flow cytometry.

Genotype	Name	Type	Country	Nuclear DNA Content (Mbp)
L1	Lira	Landrace	Portugal	3.50
L2	Lira	Landrace	Portugal	3.30
M1	Branca de Cinco Bicos	Landrace	Madeira Island, Portugal	3.26
M2	Amarela	Landrace	Madeira Island, Portugal	3.30
M3	Inglesa	Landrace	Madeira Island, Portugal	3.20
M4	São Vicente	Landrace	Madeira Island, Portugal	3.41
A1	Batata Doce Regional	Landrace	Azores Islands, Portugal	3.00
G1	Gloria	Breeding Variety	Mozambique	3.42
I5	Ininda	Breeding Variety	Mozambique	3.40
T 01		Landrace	Timor-Leste	3.23
T 05		Landrace	Timor-Leste	3.29
T 10		Landrace	Timor-Leste	3.36

A 4.80 pg medium DNA content for hexaploidy sweetpotato was suggested [48], but more recent estimates indicate a genome size of 3.12–3.29 pg/2C [37].

## Data Availability

All data are contained within this article.

## Supplementary Materials

The following supporting information can be downloaded at https://www.mdpi.com/article/10.3390/biology14111602/s1. Figure S1: DeltaK on-farm accessions; Figure S2: DeltaK genebank accessions; Table S1: Sweetpotato primer sequences; Table S2: SSR screening of 15 on-farm accessions; Table S3: SSR screening of 30 genebank accessions.

## Abbreviations

The following abbreviations are used in this manuscript:

SSR	Simple sequence repeat
PCoA	Principal coordinate analysis
PGI	Protected geographical indication
OFSP	Orange-fleshed sweetpotato
VAD	Vitamin A deficiency
CGIAR	Consultative Group on International Agricultural Research
RAPD	Random amplified polymorphic DNA
FCM	Flow cytometry
MCMC	Monte Carlo Markov chain
CIP	International Potato Center
