# Supplementary figures and images for: Genetic Diversity of Sweetpotato (Ipomoea batatas (L.) Lam.) from Portugal, Mozambique and Timor-Leste
Source: Biology (Basel). 2025 Nov 15;14(11):1602. doi: 10.3390/biology14111602 (PMC12650677; doi:10.3390/biology14111602)

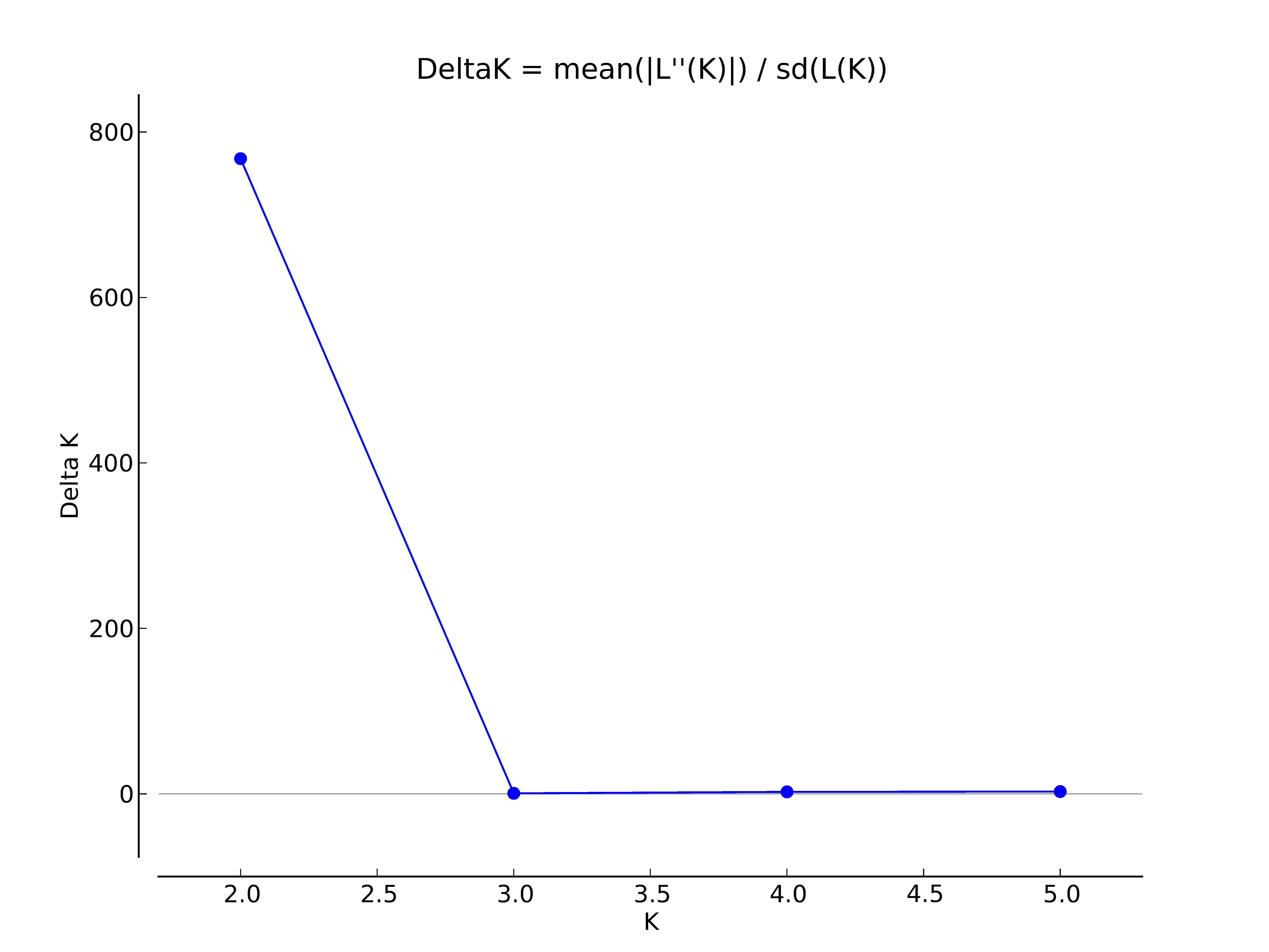

Supplement: Supplementary file 1 [file biology-14-01602-s001.zip › Figure S1 DeltaK on farm accessions-ID 3903526.jpg]

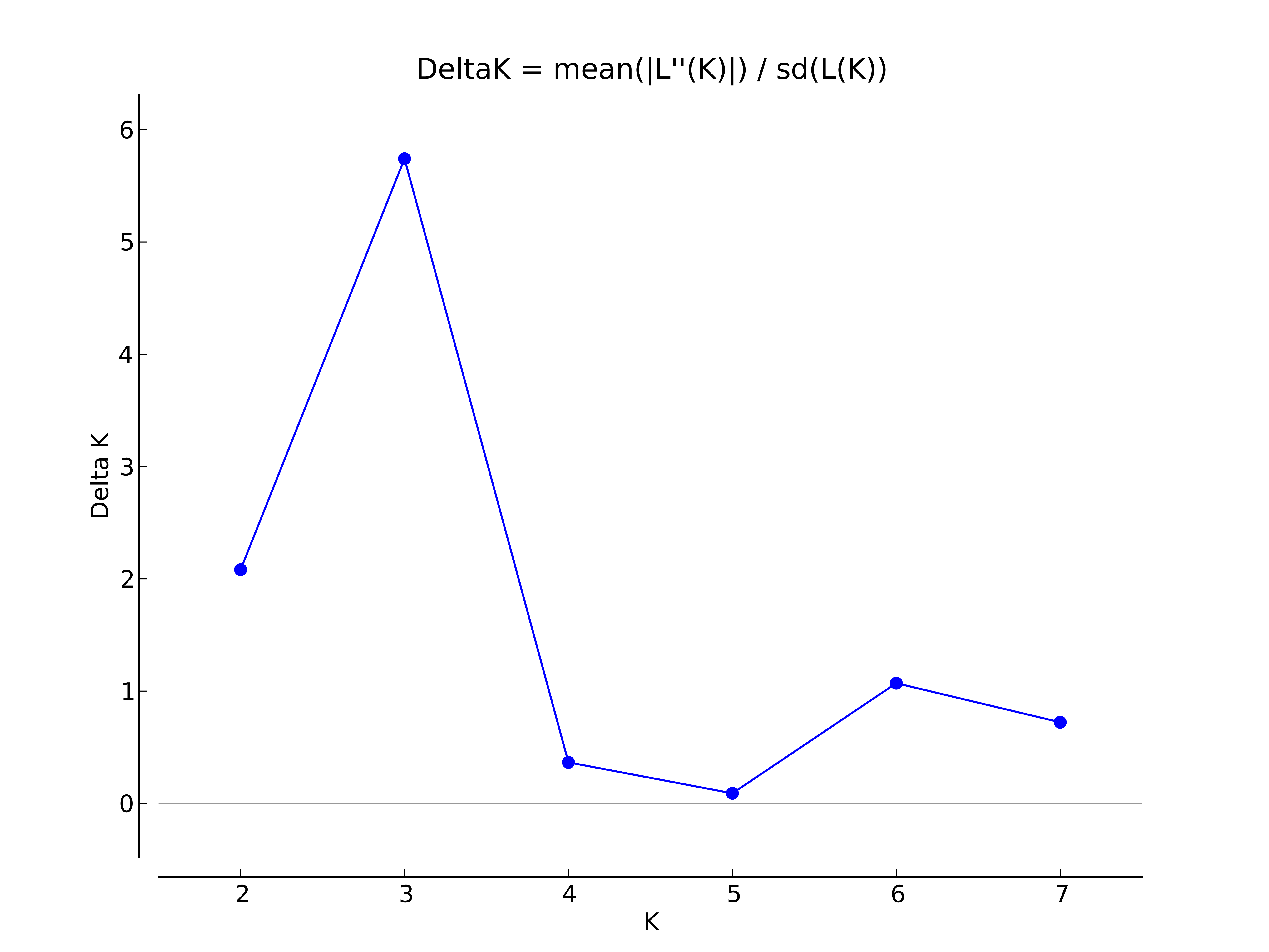

Supplement: Supplementary file 1 [file biology-14-01602-s001.zip › Figure S2 DeltaK genebank accessions-ID 3903526.jpg]
